# Engineered Probiotics for Detection and Treatment of Inflammatory Intestinal Diseases

**DOI:** 10.3389/fbioe.2020.00265

**Published:** 2020-03-31

**Authors:** Maria Barra, Tal Danino, Daniel Garrido

**Affiliations:** ^1^Department of Chemical and Bioprocess Engineering, School of Engineering, Pontificia Universidad Católica de Chile, Santiago, Chile; ^2^Department of Biomedical Engineering, Columbia University, New York, NY, United States

**Keywords:** probiotics, live biotherapeutics, biosensors, inflammatory bowel disease, intestinal inflammation, gut microbiome

## Abstract

Inflammatory intestinal diseases such as Crohn’s disease and ulcerative colitis have seen an increase in their prevalence in developing countries throughout the current decade. These are caused by a combination of genetic and environmental factors, altered immune response, intestinal epithelium disruption and dysbiosis in the gut microbiome. Current therapies are mainly focused on treating symptoms and are often expensive and ineffective in the long term. Recently, there has been an increase in our understanding of the relevance of the gut microbiome and its impact on human health. Advances in the use of probiotics and synthetic biology have led to the development of intestinal biosensors, bacteria engineered to detect inflammation biomarkers, that work as diagnostic tools. Additionally, live biotherapeutics have been engineered as delivery vehicles to produce treatment *in situ* avoiding common complications and side effects of current therapies. These genetic constructs often express a therapeutic substance constitutively, but others could be regulated externally by specific substrates, making the production of their treatment more efficient. Additionally, certain probiotics detecting specific biomarkers *in situ* and responding by generating a therapeutic substance are beginning to be developed. While most studies are still in the laboratory stage, a few modified probiotics have been tested in humans. These advances indicate that live biotherapeutics could have great potential as new treatments for inflammatory intestinal diseases.

## Introduction

The composition and function of the gut microbiome have important effects on diverse aspects of human health. The extensive network of metabolites produced by intestinal microbes can affect the integrity of the gut epithelium, energy balance and host immune responses (Matsuoka and Kanai, 2015). While certain genera are known to be dominant in the microbiomes of most adults, the diversity of bacteria that colonize the human intestine, particularly at the species level, is highly variable. A dysbiosis of the gut microbiota, the rupture of homeostasis between harmful and protective intestinal bacteria, can correlate and may be causative of certain disease states (Lakatos, 2009). These alterations have been linked to diabetes, obesity, asthma, allergy, inflammatory bowel disease (IBD), among others (Bäckhed et al., 2012).

Attempts to restore unhealthy microbiomes have been made by using probiotics. Probiotics are live microorganisms that upon consumption in adequate amounts provide beneficial effects on health (Hill et al., 2014). They have been shown to improve diseased states in the intestine, such as pouchitis, infectious diarrhea, Irritable Bowel Syndrome, *Helicobacter pylori* infection, *Clostridium difficile* infection, and antibiotic-associated diarrhea (Ritchie and Romanuk, 2012). Nevertheless, they often only transiently colonize the host and are not retained in the long term (Derrien and van Hylckama Vlieg, 2015). Additionally, current probiotics are not designed to treat a specific condition; they instead provide general health benefits. This problem raises the opportunity to use genetic engineering to develop more pragmatic probiotics that can produce substances that are relevant to treating specific conditions.

With the increased knowledge of the gut microbiome and the role of specific keystone microbes in our health, combined with the development of new synthetic biology tools, probiotic microorganisms have been engineered to diagnose and treat intestinal inflammation. These microorganisms are being designed for the sensitive and precise detection of inflammation-related biomarkers *in situ*. Besides, live biotherapeutics have been engineered with diverse functions ranging from the constitutive expression of a therapeutic substance to more complex sense/respond/record mechanisms. The aim of this review is to provide a current view of advances regarding the applications of live biotherapeutics in the diagnosis and eventual treatment of inflammatory intestinal diseases.

## Inflammatory Bowel Diseases

The two most prevalent inflammatory bowel diseases are Crohn’s disease (CD) and ulcerative colitis (UC). Both UC and CD are chronic disorders characterized by severe intestinal inflammation, but they also have significant differences. UC is characterized by the formation of superficial mucosal ulcerations and is limited to the proximity of the rectum (Xavier and Podolsky, 2007). Significant amounts of neutrophils form micro-abscesses in the lamina propria and the crypts. CD can be manifested elsewhere in the gastrointestinal tract, although the terminal ileum is most commonly affected. It is characterized by the accumulation of macrophages forming granulomas, and inflammation is usually transmural (Xavier and Podolsky, 2007). IBD symptoms could include bleeding, diarrhea, anemia, weight loss, and high levels of pain (Pithadia and Jain, 2011).

These diseases have a higher prevalence in North America and northern Europe and lower prevalence in developing countries (Baumgart and Carding, 2007). While the incidence of IBD has reached a plateau in the former, there has been a rise in the number of cases in South America, Eastern Europe, and Asia in the current decade (Burisch and Munkholm, 2013). It is estimated that approximately 6.8 million people worldwide are living with IBD (Jairath and Feagan, 2019).

The causes of IBD are believed to be multifactorial including genetic predisposition, environmental factors, alterations in the immune system, disruption in the integrity of the intestinal epithelium and dysbiosis in the gut microbiome (Matsuoka and Kanai, 2015; Martini et al., 2017). The susceptibility genes that have been identified include several pathways relevant to intestinal homeostasis. Nevertheless, these do not explain the increase in IBD cases that have been reported in developing countries suggesting the relevance of environmental factors (Khor et al., 2011). These include diet, smoking, geography and hygiene, among others (Baumgart and Carding, 2007; Lakatos, 2009). Additionally, IBD patients have been shown to have an overreactive immune system that leads to exacerbated intestinal inflammation (Baumgart and Carding, 2007). There is also a malfunctioning of the intestinal epithelium and barrier function. The epithelium acts typically as a semipermeable barrier keeping pathogens out while allowing the entrance of selective nutrients (Martini et al., 2017). It also acts as a receptor of signals from the intestinal microbiome and the immune system maintaining homeostasis. When its integrity is compromised, alterations in immune responses may occur, leading to IBD symptoms. Together, the immune system, as well as genetic and environmental factors, influence the composition of the gut microbiome, and in turn these microbes influence immune responses.

The proliferation of certain species and overproduction or lack of specific metabolites could also contribute to the development of IBD. For example, *Faecalibacterium prausnitzii* is an intestinal microbe known to have anti-inflammatory properties by secreting metabolites that block nuclear factor κB (NF-κB) and interleukin-8 (IL-8) production (Sokol et al., 2008). The numbers of this particular microbe are significantly reduced in patients with IBD (Sokol et al., 2008). Additionally, short chain fatty acids (SCFA), particularly butyrate, show protective and anti-inflammatory properties in the intestine, and they are present in lower concentrations in IBD patients (Parada Venegas et al., 2019). Therefore, studying the relevance of particular protective gut bacteria could be important for reverting dysbiosis.

Current therapies used for IBD alleviate inflammation and help to prevent flare-ups; these diseases presently have no cure (Caprilli et al., 2008). Symptoms are generally treated with corticosteroids, aminosalicylates and immunomodulators (Stein and Hanauer, 1999). Unfortunately, these drugs do not treat the cause of the disease and induce undesirable side effects, being sometimes ineffective. More recently, certain biologic treatments, usually antibodies that target specific inflammatory pathways, have been proven to be more effective. These alternatives, however, are costly and frequently delivered subcutaneously, which may increase the possibility of adverse side effects (Paramsothy et al., 2018). Another approach has been attempting to improve the composition of the gut microbiome of IBD patients through fecal microbiome transplants. This approach has been successfully used on various occasions for treating *Clostridium difficile* infection and associated diarrhea (Lopez and Grinspan, 2016). Nevertheless, this is an invasive procedure still in clinical trials with unestablished protocols and specifications, making this a riskier option (Sunkara et al., 2018). Despite receiving various treatments throughout their lives, many IBD patients eventually must undergo surgery to treat complications and alleviate symptoms (Caprilli et al., 2008). Therefore, new treatment options focusing on improving intestinal epithelium integrity rather than merely treating symptoms are necessary. Live biotherapeutics with targeted delivery and action in the intestine could be an exciting option for fulfilling the current requirements of IBD treatments.

## Biosensors

**FIGURE 1:**
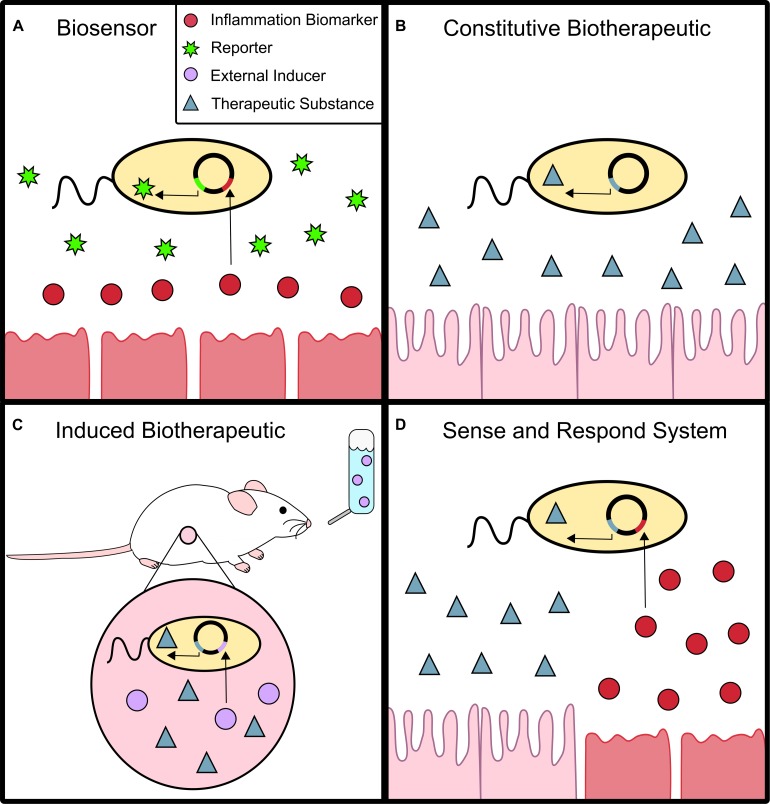
Biosensor and Live Biotherapeutics. **(A)** Biosensors can detect an inflammation biomarker which activates the expression of a reporter molecule, such as green fluorescent protein. **(B)** Constitutive biotherapeutics are probiotics that constantly produce a therapeutic substance to treat inflammation. **(C)** Induced biotherapeutics produce a therapeutic substance when activated by an external signal, commonly added to food or water. **(D)** Sense and respond systems combine biosensors and live biotherapeutics. The therapeutic substance is produced only when the probiotic detects an inflammation biomarker *in situ*.

Establishing an accurate diagnosis of gut-related diseases such as IBDs is usually difficult. First, invasive and costly procedures such as endoscopies and biopsies are normally required (Shergill et al., 2015). Second, substances indicative of disease sometimes have short half-lives or are too unstable to be easily detected. Bacterial biosensors that act *in situ* could be crucial for the future of non-invasive and precise diagnostics. Biosensors are live microorganisms engineered to detect specific biomarkers suggestive of certain disorders. Upon detection, they generate a marker that can be easily quantified, such as fluorescent proteins or colored substrates (Figure 1A).

High sensitivity and specificity toward the biomarker they recognize are important requirements for biosensors to be used as diagnostic tools. Sensitivity can be optimized by combining different genetic parts such as promoters, ribosome binding sites and terminators that confer varying strength to the output they produce. Sensitivity must also be adjusted to the biological concentrations of the molecules being sensed, which could range from pM to mM concentrations. Specificity requests the detection of the specific biomarker and no other substances with similar molecular structures that are not indicative of disease. Additionally, these must be detected in specific sections of the body and not where the biomarker is irrelevant. In order to increase specificity, biosensors can be genetically modified to sense physicochemical parameters in certain tissues, for example low oxygen tension in the intestine and tumors or certain pH. It is important for biosensors to produce a specific response exclusively when needed in order to optimize their energy resources and achieve a correct diagnosis.

The ability to optimize biosensors induced by small molecules (although not IBD biomarkers), was demonstrated in *E. coli* MG1655, DH10B, and BL21 (Meyer et al., 2019). The objective was to comply with the high standards required of biosensors including reduced promoter leakiness, high dynamic range, high sensitivity and high specificity. Different biosensors detecting relevant molecules such as 2,4-diacetylphophloroglucinol, cuminic acid, 3-oxohexanoyl-homoserine lactone, vanillic acid, isopropyl β-D-1-thiogalactopyranoside, anhydrotetracycline, L-arabinose, choline chloride, naringenin, 3,4-dihydroxybenzoic acid, sodium salicylate, and 3-hydroxytetradecanoyl-homoserine lactone were fine-tuned through directed evolution. Additionally, promoters and ribosome binding sites of varying strengths were tested in order to obtain optimal constructs. While *in vivo* studies are still required, this work shows the possibility of optimizing small molecule induction in bacteria.

Different molecules have been considered as biomarkers of gut inflammation and are used for the development of biosensors. Archer et al. (2012) used genetic parts naturally found in *Escherichia coli* to create a biosensor in the same species to detect nitric oxide (NO), a marker of intestinal inflammation (Kimura et al., 1997). NorR is a bacterial enhancer-binding protein that binds to transcription factor σ^54^ in the presence of NO, therefore activating transcription regulated by the promotor pNorV. In this biosensor pNorV regulated the expression of the DNA recombinase FimE, which activated a bidirectional circuit that in the absence of FimE (therefore, in the absence of NO) produced a yellow fluorescent protein and a cyan fluorescent protein in the presence of NO. This sensor could be an important diagnostic tool considering that NorR is highly specific toward NO and not toward other reactive oxygen species that might not necessarily be biomarkers of inflammation (Tucker et al., 2008; Bush et al., 2011).

Biosensors have also been constructed to detect thiosulfate and tetrathionate (Daeffler et al., 2017; Riglar et al., 2017). It is believed that during colitis, sulfate-reducing bacteria (mostly from the *Desulfovibrio* genus) produce hydrogen sulfide, which is converted to thiosulfate by host enzymes (Roediger et al., 1997; Levitt et al., 1999; Blachier et al., 2010; Jackson et al., 2012; Rey et al., 2013). Daeffler and colleagues computationally identified a thiosulfate sensor in *Shewanella halifaxensis* HAW-EB4 (a marine bacteria), based on a two-component system. The respective genes were cloned and optimized in *E. coli* Nissle 1917 by combining different strengths of promoters and ribosome binding sites that resulted in the best dynamic range of ligand activation. Its activation by thiosulfate was demonstrated in mice with dextran sodium sulfate (DSS)-induced inflammation (Daeffler et al., 2017). A different system has been developed to detect tetrathionate, another potential biomarker of intestinal inflammatory conditions (Riglar et al., 2017). During infection by *Salmonella typhimurium* in the mouse intestine, reactive oxygen species produced by the host convert thiosulfate to tetrathionate, which triggers inflammatory processes. Interestingly, tetrathionate is used as an alternative electron acceptor by *Salmonella*, thereby creating a niche for infection (Winter et al., 2010). Riglar and colleagues used the TtrSR two-component system from *S. Typhimurium* to create a tetrathionate biosensor in *E. coli* NGF-1, which also encoded a phage-lambda based memory circuit (Hensel et al., 1999; Riglar et al., 2017). The engineered strain was able to colonize and detect the biomarker in mice for six months under infection-induced and genetic models of inflammation. In summary, both genetic systems were highly sensitive and specific toward inflammation-triggered molecules in animal models initially making them exciting candidates for diagnosis. However, the actual relevance of thiosulfate and tetrathionate as inflammation biomarkers has not been fully studied. Particularly, tetrathionate has not been evaluated in non-mouse models due to the invasive means for its detection (Daeffler et al., 2017; Riglar et al., 2017).

There is clearly a limited knowledge regarding relevant biomarkers for gut inflammation. Recently a memory-based circuit was created to identify biosensor triggers in *E. coli* (Naydich et al., 2019). The bacteria was orally administered to healthy mice and to those with intestinal inflammation. A genetic library was created and computationally analyzed to detect these activators or repressors by comparing both conditions. Each library included a promoter and ribosome binding site, and the latter was in some cases modified to increase sensitivity to the promoter’s regulator. This is an important study considering the number of genes and operons found. However, their identity or function is not fully understood. This work provides insights to find novel biomarkers that may be indirectly related to intestinal inflammation.

Quorum sensing has also been studied as a way to detect bacterial signals and interactions in the gut. It was demonstrated that traditionally non-quorum sensing bacteria can be engineered to utilize signaling pathways to transfer information to each other in the gut (Kim et al., 2018). Native gut *E. coli* and attenuated *S. enterica* serovar Typhimurium were used as the signalers or responders. When externally induced by anhydrotetracycline, the signaler produced acyl-homoserine lactone, which was received and recorded by the responder. This system was implemented in mice and was functional throughout the gut. It could eventually be used to detect important disease biomarkers produced by pathogens and produce therapeutic substances by the responder. An example of the implementation of a quorum sensing system was developed in *L. lactis* genetically modified to detect quorum sensing signals specifically from the diarrhea-producing pathogen *Vibrio cholerae* (Mao et al., 2018). These signals activated the expression of an enzymatic reporter which was detectable in fecal samples.

Progressing toward medically applied biosensors, an ingestible probiotic and electrical based system was created to detect intestinal bleeding, wirelessly communicating the detected results to an external device (Mimee et al., 2018). *E. coli* Nissle 1917 was engineered to produce luciferase under the regulation of a synthetic promoter [P_L(HrtO__)_], which was modulated by heme-responsive repressor HrtR from *Lactococcus lactis* (Lechardeur et al., 2012). The extracellular transporter ChuA from *E. coli*, which allows diffusion of heme into the cell was also included in the circuit (Nobles et al., 2015). Therefore, heme was able to enter the cell and interact with the HrtR repressor, which liberated the P_L(HrtO)_ promoter to express luciferase. The system was able to correctly diagnose gastrointestinal bleeding in swine, also proving to be adaptable for the detection of other biomarkers and possible diagnoses of other disorders. While the system’s size, shelf-life and length of residency are factors to be improved, this innovative tool is an important example of the practicality and potential of long-studied biosensors. It represents a critical step toward fast, accurate and less invasive diagnoses.

## Live Biotherapeutics

### Constitutive Systems

Bacteria have also been engineered as delivery vehicles to produce different therapeutic substances to treat intestinal inflammation *in situ* (Figure 1B). The traditional oral or systemic delivery of many of these substances can be problematic, considering they are often unstable with short half-lives and require high doses that may cause unwanted side effects. Considering that certain bacterial strains are well suited to colonize the intestinal epithelium, live biotherapeutics have the opportunity to proliferate and simultaneously produce a desired molecule *in situ*.

Earlier attempts to develop synthetic probiotic bacteria focused on the cytokine IL-10 to reduce gut inflammation. In certain studies, the protein was expressed in genetically modified *L. lactis* (Fedorak et al., 2000; Schotte et al., 2000; Schreiber et al., 2000; Steidler et al., 2003). This lactic acid bacterium could help avoid complications presented by traditional methods of delivery, such as sensitivity to low pH and dose-dependent side effects when delivered by injection. Lactic acid bacteria have historically been used in fermented foods and are generally regarded as safe (GRAS) (del Carmen et al., 2011; Benbouziane et al., 2013). Additionally, a wide variety of genetic engineering tools have been developed for this species, and several therapeutic proteins have been produced in *L. lactis* (Benbouziane et al., 2013). Nevertheless, after a phase II clinical trial it was concluded that an IL-10 producing *L. lactis* strain was safe but ineffective at improving mucosal healing compared to a placebo (Actogenix, 2009).

More recently, the immunosuppressive cytokines IL-27 and IL-35 were expressed in *L. lactis* and non-pathogenic *E. coli*, respectively (Hanson et al., 2014; Zhang et al., 2018). IL-27 producing *L. lactis* proved more effective than both its IL-10 producing counterpart and systemic administration of IL-27 in colitis mouse models. It was shown that this strain increased the production of IL-10 in the intestinal epithelium, contributing to the effectiveness against colitis. The IL-35-producing *E. coli* not only suppressed pro-inflammatory cytokine levels, but also increased anti-inflammatory cytokine activity. Nevertheless, these mechanisms of action are not yet fully understood requiring further studies. Additionally, it would be preferable to test the construct in food-grade bacteria or in a more prominent gut microbe.

Trefoil factors (TFF) and anti-tumor necrosis factor-α (TNF-α) nanobodies (single domain antibody fragments) are other therapeutic substances that have been constitutively expressed in *L. lactis* and tested in DSS-induced colitis in mice (Vandenbroucke et al., 2004, 2010). The former are peptides that are differentially produced in specific sections in the gastrointestinal tract and have protective and reparative properties on the intestinal epithelium (Playford et al., 1996; Vandenbroucke et al., 2004). Specifically, TFF-1 and TFF-2 are produced in the stomach and duodenum in mucus-producing cells, while TFF-3 is produced in the small and large intestines, predominantly in goblet cells (Vandenbroucke et al., 2004). The peptides produced *in situ* by *L. lactis* were considerably more effective at healing colitis than the oral or rectal administration of the purified peptides (Vandenbroucke et al., 2004).

A different construct was created to counteract TNF-α (Vandenbroucke et al., 2010). It is known that levels of TNF-α are augmented in IBD patients and that this cytokine is linked to the disease’s symptoms (Vassalli, 1992; Papadakis and Targan, 2000; Adegbola et al., 2018). Antibodies for this cytokine are currently used as a treatment for IBD. Nevertheless, this treatment is expensive and can be associated with diverse systemic administration related side effects (Vandenbroucke et al., 2010). The *L. lactis* construct that produced the anti TNF-α nanobodies proved to have the beneficial effects of the aforementioned antibodies without adverse side effects.

Other possible inflammation treatments include the use of interference RNA (RNAi). Engineered *E. coli* expressing invasin and listeriolysin O are able to invade mammalian cells and therefore facilitate the transfer of genetic material (Grillot-Courvalin et al., 1998). Cyclooxygenase-2 (COX-2) is an enzyme induced by proinflammatory cytokines including TNF-α and is overexpressed in the colonic mucosa of IBD patients (Singer et al., 1998). Using the two genes previously mentioned, non-pathogenic invasive *E. coli* was engineered to transfer anti COX-2 RNAi to silence the expression of this enzyme resulting in positive effects on DSS-induced colitis in mice (Spisni et al., 2015).

While most studies utilize bacterial systems with established genetic modification systems, the cognate microorganisms are generally not dominant in the gut microbiome and their relative impact is small. This is why it is necessary to study prominent intestinal microbes as engineered probiotics for treatment of gut inflammation. For example, *Bifidobacterium longum* subsp. *longum* is a dominant microorganism found in most individuals’ microbiomes, and therefore an interesting target for delivery of biotherapeutics (Arboleya et al., 2016). This bacterium was modified to produce α-melanocyte-stimulating hormone (α-MSH), a peptide with protective and anti-inflammatory properties. α-MSH acts by increasing IL-10 and down-regulating the production of pro-inflammatory cytokines (such as TNF-α) and nitric oxide (Brzoska et al., 2010; Wei et al., 2016). The engineered strain showed significant anti-inflammatory effects in DSS-induced colitis in mice.

Other studies have focused on modifying bacteria to produce substances that counteract the action of reactive oxygen species (ROS) (Bruno-Bárcena et al., 2004; Han et al., 2006; Carroll et al., 2007; Watterlot et al., 2010; LeBlanc et al., 2011; del Carmen et al., 2014; Liu et al., 2018). Gastrointestinal tract inflammation has been associated with an overactive immune system and the accumulation of ROS. These species can damage proteins, lipids and DNA (Grisham et al., 1990; Seguí et al., 2005). ROS are usually neutralized by antioxidant enzymes, such as catalases and superoxide dismutases, which are produced *in situ* in healthy humans (Carroll et al., 2007). Therefore, increasing the production of these enzymes could improve inflammatory conditions. However, the traditional delivery of these proteins is complicated due to their short circulation half-life (Turrens et al., 1984). Ideally, they must be produced and secreted only where they are required to act, making programmable engineered probiotics an interesting option. Most studies involving ROS have genetically modified lactic acid bacteria to be used as delivery vehicles for antioxidants, achieving reduced inflammation in different *in vitro* and *in vivo* models. However, one study did utilize the more dominant bacterium, *B. longum*, to express the antioxidant enzyme manganese superoxide dismutase, reducing DSS-induced colitis in mice (Liu et al., 2018).

In a different approach, the use of a biosensor in a transgenic mouse model was combined with the production of a therapeutic peptide by intestinal epithelial cells (Breyner et al., 2017). Microbial Anti-inflammatory Molecule (MAM) is a peptide produced by *Faecalibacterium prausnitzii* that has been shown to have anti-inflammatory properties on the intestinal epithelium and was shown to block NF-κB activation *in vitro* (Quévrain et al., 2016). After failed attempts of heterologous and chemical synthesis of MAM, the authors modified *L. lactis* to carry a plasmid with MAM’s cDNA under the control of a eukaryotic promoter. The plasmid was transfected into intestinal epithelial cells, which successfully produced the peptide in a mouse model. In this model, luciferase was produced under the control of an NF-κB promoter allowing the observation of MAM’s interference with the production of the nuclear factor *in vivo*. MAM’s anti-inflammatory properties were confirmed in dinitrobenzene sulfonic acid (DNBS) and DSS mouse models.

### Inducible Systems

There are several advantages in the application of inducible probiotics instead of microorganisms producing therapeutic substances constitutively (Figure 1C). The probiotics mentioned previously have been developed using relatively simple genetic modifications, mostly expressed in a constitutive fashion and including their native transcription and translation signals. It is important to consider that constantly generating these therapeutic substances requires a large amount of energy for the probiotic. This can be detrimental for bacterial fitness where constitutive expression places a substantial cost with no major benefit. In contrast, inducible systems are easier to control and can help prevent the overproduction of the substance, which can have unknown consequences at elevated concentrations.

*Lactococcus lactis* has been engineered to produce IL-10 in a regulated manner under the control of inducible promoters. The *L. lactis* xylose inducible expression system (XIES) was used to genetically modify this same bacteria, regulating the expression of the cytokine by modifying the concentration of xylose present (del Carmen et al., 2011). This system was chosen because of its well-controlled regulation and efficiency in the production of long-term complex proteins (Miyoshi et al., 2004). The bacteria was used to ferment milk containing xylose and given to mice as a treatment for trinitrobenzenesulfonic acid (TNBS)-induced colitis. The authors hypothesized that the food matrix protected IL-10 through the gastrointestinal tract where it was able to act. Although this system is easily regulated, it is limited to the food matrix used and does not respond automatically to host signals, but rather to the concentration of xylose present.

*Bacteroides* species are dominant in the gut microbiome representing up to 25% of total microorganisms (Salyers, 1984). A xylan-inducible system has been developed in *B. ovatus* to produce human keratinocyte growth factor 2 (KGF-2; Hamady et al., 2010) and transforming growth factor β (TGF-β; Hamady et al., 2011). Both growth factors are important for maintaining intestinal integrity, being essential for epithelial cell proliferation (Barnard et al., 1989; Werner, 1998). They were both regulated by the xylanase promoter from the xylan operon in *B. ovatus*. Xylan is a fiber found in plants and is utilized by certain gut bacteria, including *B. ovatus* (Hespell and Whitehead, 1990; Thomson, 1993). The fiber was added to the experimental mice’s food and water to induce the expression of the therapeutic substances. Using this bacterium as a delivery system has several advantages. It is an important anaerobic human gut commensal and certain *Bacteroides* have been found in the intestinal mucin layer (Croucher et al., 1983). Therefore, its survival and location of action are safer and more specific than other potential probiotics such as *L. lactis*. Both constructs were easily regulated and showed improved colitis conditions in mice.

Recently, *E. coli* Nissle 1917 was engineered to produce an extracellular matrix containing all three trefoil factors to treat inflammation and help re-build the intestinal epithelium (Praveschotinunt et al., 2019). CsgA is the monomer unit for the curli fibers that make up the fibrous matrix. The trefoil factors were fused to the CsgA C-terminus. Along with other genes required for the assembly and secretion of the modified polymer, they were placed under the control of an arabinose-induced promoter and incorporated into a plasmid with kanamycin resistance. The modified probiotic was tested in mice with DSS-induced colitis. In order to induce expression and assure plasmid stability the mice were given water infused with kanamycin and arabinose. The biotherapeutic improved inflammation, intestinal epithelium integrity and correlated with a decrease in the production of inflammatory cytokines and enzymes. While this is an interesting combination of delivery technique and therapeutic action, the authors note the necessity of replacing antibiotic-based selection and using induction based on environmental signals rather than an external substance.

### Sense and Respond Systems

With the objective of creating more specific, efficient and well-regulated live biotherapeutics, sense and respond genetic mechanisms are beginning to be developed. These systems are a more specific type of inducible probiotic, considering that their objective is to respond to the state of a certain organ and/or to a disease biomarker, rather than to an external activation source (Figure 1D). For example, a method that has been used to regulate IL-10 expression is through a stress-induced system (Benbouziane et al., 2013). Benbouziane and colleagues used the *L. lactis* groESL operon promoter, which has been shown to respond to low pH, heat shock and UV-radiation *in vitro* (Arnau et al., 1996; Hartke et al., 1997). Its activation has also been reported *in vivo* in a murine model (Roy et al., 2008). This system increases the chances that the therapeutic substance is produced *in situ* considering that the administered bacteria automatically goes through stressful conditions compared with laboratory growth conditions. Nevertheless, this circuit is not specific to gut inflammation conditions, and several stresses could be found along the gastrointestinal tract.

Recently, a genetic circuit based on NO detection, pseudotaxis and secretion was created in *E. coli* to produce and export granulocyte macrophage-colony stimulating factor (GM-CSF) *in situ* (McKay et al., 2018). NO activated the expression of CheZ, a motility regulator protein in an *E. coli* strain otherwise lacking this gene. Because NO acted as an attractor, the modified bacteria moved toward higher concentrations of this biomarker, where inflammation should be more prevalent. GM-CSF was chosen because it is reported to have therapeutic effects in patients with Crohn’s disease by helping restore the mucosal barrier, stimulate neutrophils and sensitize pathogenic bacteria (Dieckgraefe and Korzenik, 2002; Korzenik et al., 2005; Choudhary et al., 2015). Its extracellular release was allowed through the inclusion of *tolAIII*, the gene of a pore-forming protein, in the genetic circuit. The release was confirmed with higher concentrations of GM-CSF in the supernatant of bacteria that included *tolAIII*. This engineered probiotic can respond directly to environmental inflammation conditions but must be tested *in vivo* in order to evaluate its therapeutic potential for IBD patients.

Another sense and respond system was engineered to treat *Salmonella* infection. As mentioned previously, *Salmonella typhimurium* can utilize tetrathionate as an electron acceptor creating a niche for infection and producing gut inflammation (Riglar et al., 2017). The tetrathionate biosensor system was incorporated into a plasmid controlling the expression of microcin H47 and transformed into *E. coli* Nissle 1917 (Palmer et al., 2018). Microcin H47 is a peptide originally obtained from *E. coli* H47 that was confirmed to inhibit growth of *Salmonella in vitro*. The modified strain of *E. coli* Nissle effectively produced the microcin in the presence of tetrathionate inhibiting *Salmonella* growth *in vitro*. While this is an interesting example of a sense and respond construct, it must also be tested *in vivo* before extrapolating further conclusions about its therapeutic effectiveness.

In theory, sense and respond systems have a higher impact and are more beneficial for inflammation treatment than simpler constructs. Nevertheless, this also implies more extensive genetic modifications that can weaken the circuit’s durability and bacterial fitness. Still, because so few of these types of circuits have been developed, they should be expanded upon and tested in mice. It is important to quickly confirm whether they are more effective as treatment in order to validate or reject their further development.

## Challenges Regarding Live Biotherapeutics

While there is great potential in using engineered bacteria as diagnostic tools and live biotherapeutics, there is much left to improve and achieve. Although most biosensor and live biotherapeutic studies have focused on modifying *L. lactis* and *E. coli*, it is essential to develop new genetic engineering tools in more dominant microorganisms from the gut microbiome. These species may be more effective in treating gut inflammation since they have several adaptations for colonization of the human intestine, safe interaction with the immune system and reach high cell numbers, therefore, increasing the rate of success. Nevertheless, these species are usually anaerobic, more difficult to manipulate and their efficacy as genetically modified probiotics is strain-dependent. Additionally, in the gut microbiome introduced engineered bacteria could be more prone to genetic mutations, loss of therapeutic functions and decreased growth rates due to the energetic burden caused by synthetic circuits. Barriers including the lack of reliable genetic engineering tools for these species and methods for their encapsulation and delivery must be overcome.

Safety issues regarding containment, specificity and toxicity issues also arise. An engineered bacteria ingested by a person must be contained within that person and unable to transfer its modified DNA to the environment where it could have unpredicted consequences. Bacterial kill switches and quorum sensing-based autolysis systems have been incorporated into genetic circuits in order to avoid these issues (van de Poel and Robaey, 2017; Chowdhury et al., 2019). It is also fundamental for the biotherapeutic to be secreted directly to its target. In this sense, detecting biomarkers is not only important for diagnosing gut inflammation, but also for correctly generating treatment. This will allow the sidestepping of the substance acting in an incorrect organ and high doses that can have side effects. Additionally, the engineered probiotics must be toxin-free in order to avoid creating more damage than benefits, which is why food-grade bacteria initially seem appealing. A hesitant reception may be generated toward the use of new and generally unheard of gut-relevant probiotics.

It may be challenging to achieve colonization of individual bacteria through probiotics due to the difficulty of finding a niche to survive in the gut microbiome. The design of microbial consortia could be useful for this purpose. This would ideally depend on an individual IBD patient’s needs regarding the cause of his or her symptoms. For example, genetically modified SCFA-producing live biotherapeutics could eventually contribute to maintaining a balanced and healthy microbiome. Additionally, if multiple complementing species were to be ingested simultaneously, their chances of survival could improve. Further, different parts of a genetic circuit could be distributed among different species, increasing their co-dependence and relieving the metabolic strain on each one. The concept of personalized consortia is a future possibility that will depend on medical validity, safety and the future of personalized medicine.

## Conclusion

Current advances in developing live biotherapeutics indicate they are a promising treatment for IBD. With synthetic biology tools, scientists have the ability to rapidly create various genetic circuits at a time and by high throughput screening, multitudes of options can be evaluated. The best candidates should then go on to clinical trials. Having a variety of options may be essential in the future of personalized medicine in which an individual’s symptoms could be treated in a specific manner. This is crucial considering the diversity of the human gut microbiome and the plethora of possible causes that generate IBD. Additionally, the treatment would be non-invasive, direct and fast. Depending on the species and the therapeutic substance used, they could help restore normal microbiome conditions and heal the intestinal epithelium rather than simply treat recurring symptoms.

## Author Contributions

TD and DG conceived the manuscript and reviewed the last version. MB and DG wrote the manuscript.

## Conflict of Interest

The authors declare that the research was conducted in the absence of any commercial or financial relationships that could be construed as a potential conflict of interest.
